# Neutrophil Extracellular Traps and Their Possible Implications in Ocular Herpes Infection

**DOI:** 10.3390/pathogens12020209

**Published:** 2023-01-29

**Authors:** Divya Kapoor, Deepak Shukla

**Affiliations:** 1 Department of Ophthalmology and Visual Sciences, College of Medicine, University of Illinois at Chicago, 1905 W. Taylor St., Chicago, IL 60612, USA; 2 Department of Microbiology and Immunology, College of Medicine, University of Illinois at Chicago, 835 S. Wolcott, Chicago, IL 60612, USA

**Keywords:** NETosis, herpes ocular infection, survival NETosis, lytic NETosis

## Abstract

Neutrophil extracellular traps (NETs) are net-like structures released from neutrophils. NETs predominantly contain cell-free deoxyribonucleic acid (DNA) decorated with histones and neutrophil granule proteins. Numerous extrinsic and intrinsic stimuli can induce the formation of NETs such as pathogens, cytokines, immune complexes, microcrystals, antibodies, and other physiological stimuli. The mechanism of NETosis induction can either be ROS-dependent or independent based on the catalase producing activity of the pathogen. NADPH is the source of ROS production, which in turn depends on the upregulation of Ca2+ production in the cytoplasm. ROS-independent induction of NETosis is regulated through toll-like receptors (TLRs). Besides capturing and eliminating pathogens, NETs also aggravate the inflammatory response and thus act as a double-edged sword. Currently, there are growing reports of NETosis induction during bacterial and fungal ocular infections leading to different pathologies, but there is no direct report suggesting its role during herpes simplex virus (HSV) infection. There are innumerable independent reports showing that the major effectors of NETosis are also directly affected by HSV infection, and thus, there is a strong possibility that HSV interacts with these facilitators that can either result in virally mediated modulation of NETosis or NETosis-mediated suppression of ocular HSV infection. This review focuses on the mechanism of NETs formation during different ocular pathologies, with its prime focus on highlighting their potential implications during HSV ocular infections and acting as prospective targets for the treatment of ocular diseases.

## 1. Introduction

Herpesviruses represent a large family of DNA viruses. They are characterized into α-, β-, or γ-subfamilies based on their biological and genetic similarities. All members are known to cause lytic infections and latency in definite cells. Among the identified herpesviruses, eight are known to infect humans. Ocular herpes infection is majorly caused by herpes simplex virus type 1 (HSV-1) and occasionally by herpes simplex virus type 2 (HSV-2). Both HSV-1 and HSV-2 are α-herpesviruses for which more than 65% of the population is found to be seropositive in the USA. HSV causes different corneal complications such as epithelial keratitis or stromal keratitis, which can lead to diverse ocular manifestations such as blepharitis, canalicular obstruction, conjunctivitis, iritis, and retinitis [1].

Upon infection, HSV-1 first replicates in corneal epithelium and then spreads to the trigeminal ganglion (TG) for establishment of latency. The initial infection causes an induction of immune reaction. The host mounts both innate and adaptive immunological control of herpesvirus, with B- and T cells having role in the adaptive immune response. Post-infection innate immune response is the first line of defense, with type I interferons (IFNs) and natural killer (NK) cells having crucial roles in repression of the infection [2]. The innate immune system is triggered, ensuing the detection of infection by the pattern recognition receptors (PRRs), mainly toll-like receptors (TLRs). There are different TLRs in the host cell based on their location in the plasma membrane or endosomal compartments. The recognition of pathogen-associated molecular patterns (PAMPs) by PRRs leads to induction of intracellular signaling events stimulating the production of pro-inflammatory cells, chemokines, and cytokines including type I interferons that progressively infiltrate into the stroma. The inflammatory cells composed of neutrophils, dendritic cells (DCs), natural killer (NK) cells, and macrophages help clear the virus during early corneal infection. Multiple downstream effects of PRRs activation lead to induction of cytokines as well as multiple programmed cell death pathways [3]. For instance, herpes can be detected by intracellular DNA-sensing proteins such as IFNγ-inducible protein 16 (IFI16) that can further activate IRF3 and NF-κB pathways along with caspase 2 and caspase 3 stimulation (apoptotic markers) [4]. Such actions posed by the host challenge HSV-1 productive infection, as the virus needs to evade host immune response and also keep the cell alive and functioning.

As a successful host-adapted virus, HSV-1 has evolved with the host cell to impose diverse immune-evasion strategies. However, HSV-1 can be recognized by the innate immune system effectively. Neutrophils are the most abundant innate immune cells in the blood and are at the forefront during an ocular infection [5]. Recruitment of neutrophils at the ocular site of infection triggers a cascade of effector functions that are well reported in combating the recurrent bacterial, fungal, and viral infections. This effect is also reported by clinicians as the change in neutrophil numbers during an early infection [6,7,8]. However, the focus of this review article is on neutrophil extracellular traps (NETs) that are generated in response to infection. Brinkmann et al. [9] were the original group to demonstrate the release of NETs upon activation that leads to entrapment of Gram-positive and -negative bacteria. These traps that are composed of granule proteins and chromatin degrade virulence factors and kill bacteria. Further, the fabrication and release of these extracellular traps was described as NETosis, which was used as a suicidal tool by neutrophils to kill bacteria. After this discovery, multiple researchers observed the phenomenon of NETosis under multiple pathologies such as cancer, viral, bacterial, protozoal, and fungal infections. Additionally, there is growing evidence of ETosis, which is a general term depicting extracellular traps formed by multiple immune cells such as neutrophils, monocytes, and macrophages that act as physical barriers for bacteria, viruses, and fungi [10,11]. The different ETs have numerous features in common irrespective of the type of cells from which they are released, including a DNA backbone with embedded antimicrobial peptides, proteases, and histones. However, they also demonstrate preeminent individual differences such as the type of sub-cellular compartments from where the DNA backbone originates (e.g., nucleus or mitochondria), the distribution of responding cells within the pool, and/or the molecular mechanism/s underlying the ETs formation [12]. However, despite the fact that neutrophils are the first line of defense, there is still no report of NETosis in herpes infection. Here, we analyze the current knowledge of the NETosis in ocular infections by various pathogens and raise the strong possibility of the phenomenon happening during an ocular HSV-1 infection.

## 2. Mechanism of NETosis

The word “osis” in the term NETosis depicts death that implies the loss of the pathogen entrapped in the NET, but it remains debatable whether the NET release is an active and explicit biological outcome of the host response or simply a result of cellular burst due to accumulation of membrane permeable toxins or stress molecules due to an infection. Further highlighting the complex nature of this phenomenon, experts of cell death pathways are unsure about how the active NET release is related to other known programmed cell death pathways such as apoptosis, necroptosis, and pyroptosis. NETosis was initially defined as a suicidal gizmo to trap and kill bacteria extracellularly, but new reports show that NET release can be triggered by numerous other pathogenic (fungi, viruses, and parasites) and non-pathogenic (PMA, Ionomycin, LPS) stimulants [13]. Reportedly, NETosis is also involved in the progression of immune-facilitated disorders. Thus, understanding different mechanisms of NETosis is indispensable to comprehend neutrophil-driven infection and/or inflammatory diseases.

### 2.1. Suicidal/Lytic NETosis

The formation of NETs is a lesser explored type of cell death that necessitates nuclear envelope disintegration and chromatin decondensation. Upon induction of NETosis, the cell membrane ruptures, and decondensed chromatin releases its granular matter into the extracellular space, leading to the dissolution of plasmatic membrane, ultimately causing neutrophil death. Subsequently, these NETs can entangle different pathogens such as bacteria, fungi, protozoa, and viruses. Using imaging experimentation as a major tool, Fuchs et al. assigned NET formation as the final step of active neutrophil death in response to phorbol ester and *Staphylococcus aureus* [14]. Essentially, this form of cell death allows the complete release of chromatin into the extracellular space without any DNA fragmentation. The detailed cellular mechanism is still under research, but the key elements of lytic NETosis are well defined and constitute neutrophil elastase (NE) and myeloperoxidase (MPO), both of which form the part of primary neutrophilic granules. The reported mechanism of lytic NETosis associates reactive oxygen species (ROS) formation to NET release through an NE-mediated process. ROS generated by NADPH oxidase stimulates NE translocation from cytoplasmic granules to the nucleus, where it cuts histones and promotes the chromatin unfolding and degradation of the nuclear membrane. MPO also synergizes with the NE in DNA decondensation and triggers NET independent of its enzymatic activity, suggesting the complex nature of NETosis [15]. 

Ligation of different pathogens or immune crystals triggers the induction of ROS via MEK–extracellular-signal-regulated kinase (ERK) signaling pathway that further stimulates an MPO-NE pathway. Additionally, Wang laboratory described the role of peptidylarginine deiminase 4 (PAD4) in histone citrullination, heterochromatin decondensation, and NET formation and thus its crucial role in innate immunity [16,17,18]. Upon stimulation of divalent calcium ion (Ca^2+^), PAD4 can reduce the positively charged histones, which transform histone arginines to citrullines. After this stimulation, nicotinamide adenine dinucleotide phosphate (NADPH) oxidase advances to ROS generation, causing the catalyzation of superoxide dismutase (SOD) to produce hydrogen peroxide (H_2_O_2_). The H_2_O_2_ then interacts with MPO to produce hypochlorous acid (HOCl) that leads to chlorination of histones and loosens the histone–DNA interactions, similar to histone citrullination [19]. Furthermore, reports suggest that Raf-1 proto-oncogene serine/threonine kinase (c-Raf), mitogen-activated protein kinase (MEK), protein kinase B (Akt), extracellular signal-regulated kinase (ERK), and PKC pathways are upstream to NADPH oxidase production and involved in lytic NETosis [20,21]. Interestingly, the whole c-Raf-MEK-ERK pathway completes in 2–4 h. Additionally, PMA, ionomycin, concanavalin A, bacteria, fungi, and cytokines such as IL-6 and Il-8 are strong inducers of NADPH oxidase-mediated NETosis [7,22,23].

#### 2.1.1. HSV and Suicidal NETosis

**Figure 1:**
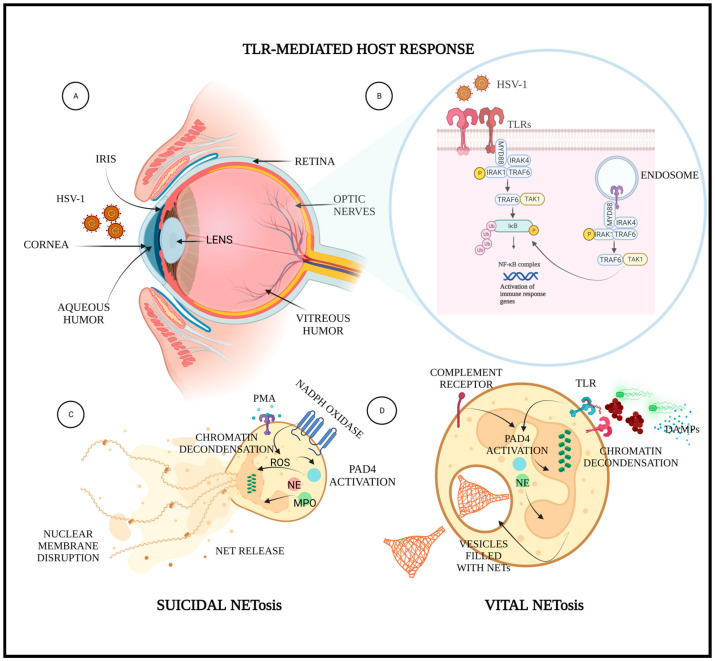
Schematic showing HSV-1 ocular infection, its recognition by the TLRs on corneal epithelial cells, and mechanisms associated with suicidal and vital NETosis. Implication of NETosis during an ocular HSV-1 infection: (**A**) sagittal view of an eye infected with HSV-1; (**B**) zoomed out description of host innate immune response against ocular HSV-1 infection; (**C**) suicidal NETosis showing ROS-dependent lytic NETosis; (**D**) lytic NETosis showing TLR-dependent non-lytic NETosis. Created in bioRender.

HSV evades host immune responses to establish a successful lytic infection. It protects its clearance from the immune system by a number of mechanisms such as inhibition of interferon response; evasion of complement-mediated destruction by expressing glycoprotein C, which binds to the C3b complement component; inhibition of autophagy by neurovirulence protein ICP34.5; and suppression of the cGAS–STING signaling pathway by HSV–1 protein UL41 and VP22 [24]. In addition, to ensure a lifelong infection, HSV employs diverse molecular approaches to escape host cell death responses. For instance, the viral UL39-encoded viral protein ICP6 suppresses both caspase-8 and RHIM-dependent RIPK3 activities in host cells [25]. Similarly, HSV-1 ICP27 inhibits GSDME-mediated pyroptosis for enhancing viral replication in host cells [26]. Ironically, no reports claim any correlation between HSV infection and NETosis or herpes-mediated modulation of NETosis. It is noteworthy that HSV is known to modulate or affect the pathways that find involvement in suicidal NETosis. Thus, herpes-mediated modulation of such pathways puts forward the possibility of the virally mediated modulation of NEtosis shown in Figure 1.

#### 2.1.2. Reactive Oxygen Species: In Milieu with NETosis and HSV Infection

ROS are considered essential for NETs formation. ROS generation is a consequence of the activation of the NADPH oxidase (NOX) family of enzymes. NOX-dependent NETosis agonists such as PMA and LPS induce the generation of massive amounts of ROS in neutrophils. High concentrations of ROS and antimicrobial peptides render antimicrobial activity to neutrophil-generated phagosomes. The pharmacological inhibition of NADPH oxidase enzyme by diphenylene iodonium abrogates the NET formation, ROS production, and ultimately leads to cell death in neutrophils that were pretreated with inducers of NETosis. Furthermore, patients with chronic granulomatosis, who have a genetically defective NADPH oxidase enzyme, do not produce NETs [27]. Thus, the levels of ROS in neutrophils critically governs the cell death, i.e., NETosis [14].

Coincidentally, HSV also induces NADPH oxidase-dependent ROS generation in infected cells. In cultured cells, the increase in ROS levels is detected as early as 1 h post infection [28]. The maintenance of an ROS-mediated mild oxidative stress is thought to facilitate replication and pathogenicity of herpes viruses. The supplementation of antioxidants leads to a reduction in the viral load, indicating that replication is favored by a state of oxidative stress or ROS production [29]. Treatment with low concentrations of oxidative stress inducers, for instance, 4-HNE, aids in viral replication, whereas increase in concentration beyond a specific level inhibits the viral replication [28]. Under a productive HSV replication, the levels of ROS generated by HSV infection are known to impair the interferon response by oxidizing Cysteine 147 on murine STING, which is analogous to Cysteine 148 of human STING [30]. However, ROS are known to trigger the phenomenon of cell death only at higher levels where the cell’s antioxidant mechanisms fail [31]. This might be the possible reason for inhibition of HSV replication upon treatment with higher concentrations of ROS inducers such as 4-HNE. At lower levels, ROS are involved in different signaling pathways [32]. Thereby, they are known to aid HSV replication in a productive infection by suppressing host immune responses. Therefore, it seems that the levels of ROS generated in the HSV-infected cells are insufficient for triggering NETosis. While the induction of different forms of cell deaths curb HSV infection, HSV infection-triggered induction of ROS promotes viral replication, possibly via the suppression of different cell death pathways. However, at the peak of infection, the ROS levels required for NETosis may be achieved, a possibility that requires additional scrutiny. 

### 2.2. Live Cell/Vital NETosis

Initially, NETs formation was reported as an oxidant-dependent event that leads to lysis of neutrophils. Recently, Pilsczek et al. described a non-lytic mechanism of NETs formation, where neutrophils responded uniquely to *Staphylococcus aureus* infection. In this form of NETosis, the nucleus of neutrophil condenses and becomes round [33]. Then, there is partition of the inner and outer nuclear membranes and budding of vesicles that are filled with nuclear DNA. This marks the expulsion of vesicles from the cell, where they burst and release chromatin. Thus, the whole process occurs swiftly and in an oxidant-independent manner in 5–60 min. Unlike lytic NETosis, vital NETosis contains a very little amount of mitochondrial DNA. Lytic NETosis has a limited amount of proteolytic activity but enough to trap and kill *S. aureus*. 

The lytic form of NETosis is established on the phenomenon of neutrophil death; however, it leaves certain questions unaddressed and creates a confusion regarding how the obligatory events of NETosis such as chemotaxis and phagocytosis are carried out by a dead neutrophil. One possibility is that a PMN could initially perform its live cell functions and degrade intracellular pathogens prior to its death and then trap extracellular pathogens after its lytic NETosis. This constitutes a progressive model of live cell functions advanced by suicidal cell functions. Another possibility exists that demarcates the population of neutrophils into two subsets. One set of neutrophils may lead to live cell functions, whereas another set could lead to lytic form of NETosis. This would suggest that traditional known functions of NETosis and lytic NETosis are mutually exclusive events. To date, inadequate evidence is present to encourage this hypothesis. An additional hypothesis persists in the field that particular subsets of neutrophils endure the NETosis process and persist to execute the tasks necessary to identify, seize, and kill pathogens. This is the most accepted idea given that at most 20% to 25% of PMNs release NETs. This view was also supported by Clark et al., as they demonstrated the NET release from an intact neutrophil [34]. In addition, Yousefi et al. demonstrated the similar phenomenon in eosinophils as well as neutrophils [35].

The fundamental difference between lytic and vital NETosis other than time of NET release is the nature of stimulation. For example, suicidal NETosis has mostly been exhibited by chemical stimulants. In contrast, vital NETosis has been shown after PRRs recognition by the host. For instance, LPS, a Gram-negative bacterial stimulus, promotes quick, non-lytic NET release. This rapid NETosis was TLR4-mediated on platelets that accelerated PMN activation. Stimulation by a Gram-positive bacteria in vivo also leads to vital NETosis via both TLR2 and the complement system [36]. Thus, activation of the vital NETosis pathway has been reported against multiple groups of microbial pathogens. For instance, a recent report found that *Candida albicans* promoted NETosis within 30 min in a fibronectin- and complement-dependent manner [37].

#### Toll-Like Receptors: In Milieu with NETosis and HSV Infection

The invading pathogens are often detected by PRRs for instigating immune responses. Toll-like receptors are known to play vital role in recognition of pathogens and induction of NETosis [38]. Human neutrophils are known to possess all TLRs except TLR3, where different TLR is employed to recognize a different pathogen. For instance, TLR2 signaling pathway is employed to induce NET release against *Mycoplasma agalactiae* [39], whereas TLR2 and TLR4 are vital for ROS-dependent NETosis initiation during *Fonsecaea pedrosoi* infection [40]. Similarly, TLR7 and TLR8 recognize human immunodeficiency virus 1 (HIV) nucleic acid and trigger the induction of NETosis [22], whereas chikungunya virus (CHIKV) is captured by TLR7-elicited, ROS-dependent NETosis [41]. In addition, mitochondrial DNA (mtDNA) is also known to activate neutrophils via the cyclic GMP-AMP synthase (cGAS) and TLR9 pathways to stimulate NETosis. 

Different TLRs are also involved in recognition of HSV by the host immune system. HSV is recognized by TLR2 on the cell membrane, probably in conjunction with TLR1. Different reports have claimed that TLR2, TLR3, TLR4, and TLR9 are capable of detecting definite proteins of HSV such as glycoprotein B (gB), glycoprotein H (gH), glycoprotein K (gK), glycoprotein L (gL), and US2 protein in the activation and reactivation of HSV [42]. The dual TLR2/9 recognition has been reported as vital to fight against mucosal HSV infection. The dual ablation of TLR2/9 has been reported in high mortality rates as compared with TLR2 or TLR9 deficiency alone, overlapping with aggravated viral load in central nervous system tissues [39]. Similarly, TLR3 is required to control HSV in the central nervous system [43]. Although there are no published data on TLRs-mediated NETosis for killing HSV-1, it is obvious that TLRs play a crucial role in combatting HSV; thereby, here arises a possibility that TLRs-mediated antiviral response may be partially warranted by TLRs-trigged NETosis, as shown in Figure 1.

## 3. HSV-1, Neutrophils, and Eye

The herpes-mediated ocular infection is caused by both HSV type 1 and type 2 viruses. These neurotropic viruses affect the whole eye [44,45,46]. HSV keratitis is the most common corneal blindness in developing nations, affecting 60–95% of adults around the globe. USA accounts for 400,000 cases of HSV keratitis, with 58,000/year of recurrent infections and 24,000 new cases every year [47].

Neutrophils are commonly deemed to engage in a positive role in host defense. They are strikingly heightened in the tissues during any infection, and any dropdown in their numbers is known to worsen the pathology. For instance, neutrophil reduction ensued in aggravated viral loads and mortality in murine models of neurotropic mouse hepatitis virus infection. However, improper and sustained neutrophil stimulation can also result in damaging effects to the host, including acute illness such as pneumonia and acute respiratory distress syndrome [48]. However, the status of the cornea as the immune-privileged organ provides an additional benefit by precluding the possibility of cytokine storm and neutrophil-associated inflammation and cell damage. 

Neutrophils attracted to the lungs of diseased animals produce proinflammatory mediators and toxic elements (e.g., cytokines, defensins, peroxidases, hydrolytic enzymes, and ROS) that can stimulate pathological characteristics. Moreover, using the lipid mediator resolvin E1 and reducing the neutrophil inflow during an ocular HSV infection further reduces the severity of stromal keratitis lesions in humans [49]. In contrast, intensified neutrophil infiltration and no IL-10 production led to lethal murine cytomegalovirus (MCMV) brain infection [50]. Improved mortality rate in virus-infected elderly patients was associated with enhanced IL-17A production, which further intensified neutrophil function and triggered liver damage. Consequently, it was lethal in mice infected with either HSV-2 or MCMV. It is therefore important to thoroughly scrutinize the characteristics of the neutrophil role vital for a stable antiviral response to acquire novel treatments against dreadful ocular HSV infections.

## 4. NETosis as Host Defense on Ocular Surface

The ocular surface is exposed constantly to the outer environment and thus acts as a direct invading site for multiple pathogens. Further, the activity of immune cells is also suppressed in the ocular microenvironment due to release of immunomodulators, which is composed of cytokines, growth factors, neuropeptides, and soluble receptors. Thus, the immune privilege of the ocular site mediates the activation of antigen-specific regulatory immunity [51]. During ocular infection, neutrophils are recruited as the first line of innate immune defense by the host. Subsequently, there is growing evidence that NETs formation by neutrophils exhibits a broad range of antimicrobial activity against a range of bacteria, fungi, parasites, and viruses that are responsible for ocular infections. NETs formation by neutrophils kill the microbes by immobilizing the pathogens, but there are some controversial results showing NETs clumps with microbes without any dead bacteria [52]. The experts suggests that the skepticism is due to difference in the techniques employed to assess the killed microbes quantitatively. However, there is ample evidence supporting the destroying ability of NETs against a broad array of microbes, especially during an ocular infection, which is discussed below.

### 4.1. NETS in Bacterial Eye Diseases

Neutrophils can kill the pathogenic bacteria by phagocytosis, degranulation, and/or NETs formation as the front line of host defense [53,54]. Many reports suggest that NETs formation by neutrophils protects against corneal infection by *Pseudomonas* and *Aspergillus* on the ocular surface [55,56,57]. *Pseudomonas aeruginosa* forms biofilm, which is a prominent source of bacterial keratitis with aggressive and swiftly developing attributes. Biofilm is composed of a type-3 secretion system (T3SS) that produces numerous virulence factors such as ExoS, ExoT, ExoY, and ExoU and inserts these into the host cells along with Psl exopolysaccharide, which leads to formation of biofilms. The biofilm formation attracts neutrophils extensively, specifically due to elevated accumulation of T3SS, followed by NET release that finally confines the bacteria to a dead zone of DNA and degraded extracellular matrix (ECM). 

This aids to constrain bacteria to the outer ocular surface and impedes the spreading of the bacteria to other major organs, especially the brain [55]. Recently, researchers showed that inhibiting ROS could be an efficient treatment of bacterial keratitis caused by *P. aeruginosa*. They employed a small peptide comprised of 43 amino acids with thymosin b4 (Tb4) as an adjunct that precisely reduces the permeation of polymorphonuclear leukocyte (PMNs), up-regulates the anti-inflammatory markers, obstructs ROS generation both in vivo and in vitro, down-regulates NETs, and thus regulates neutrophils apoptosis [58].

Another ocular infection causing bacteria is *S. aureus*, which produces numerable toxins and enzymes that can potentially lead to permanent vision loss. *S. aureus* have been reported as the primary pathogen causing blepharitis, conjunctivitis, and keratitis in 47.6%, 26.6%, and 25% of patients [59]. Vision loss during a bacterial infection is attributed to dysfunctional inflammation that leads to host-induced inflammatory damage. Many studies have reported the upregulation of neutrophils as a predictor of acute bacterial keratitis [60,61,62]. Multiple strains of *S. aureus* produce diverse types of toxins, namely α-toxin and β-toxin. *S. aureus* 8325-4 is a α-toxin-positive parent strain that causes a devastating epithelial infection by initially disrupting the epithelial cell barriers in the cornea, ultimately causing the cell lysis that leads to exposed stroma for further evading pathogenic bacteria. Specifically, the neutrophil count increases exponentially at the site of stromal infection, and thus, α-toxin is identified as a prominent virulence factor during *S.-aureus*-induced keratitis [63], whereas β-toxin is a type of sphingomyelinase and only acts as a facilitator of edema and does not show extensive ocular damage. Additionally, many clinicians use the movement of permeating PMNs to evaluate ocular pathology during bacterial keratitis [64].

There is enough evidence suggesting the NETosis inducing abilities of *S. aureus*, but more intriguing reports illustrate the evolving mechanisms to elude its NET killing [9,14]. One approach entails the production of pore-forming virulence factors that lead to neutrophil neutralization and stimulate necrosis. Furthermore, catalase production by *S. aureus* blocks the H_2_O_2_ production during lytic NETosis and shields itself from oxidation and blocks NETosis completely. Consequently, the neutrophils may have advanced to their secondary and swift, vital NETosis mechanism, which is independent of ROS, and this way, the host is able to constrain catalase-positive bacterial strains [14]. 

### 4.2. NETs in Fungal Eye Diseaes

NETs produced by neutrophils play a vital role in restricting fungal infections. However, only a few fungal species are known to stimulate hosts for NETs production and be effectively eradicated by them. *C. albicans* (yeast) is the most studied fungal pathogen involved in inducing NET production, and interestingly, it is infamous for causing fungal keratitis along with other filamentous fungi (*Fusarium* and *Aspergillus*). *C. albicans* is the most common asymptomatic colonizer of oral, mucosal, and ocular surfaces and is found in 30–50% of the population. Prominently, it is more devastating, as it is a prevalent opportunistic pathogen in immunocompromised patients, causing a 40% mortality rate [65]. Its strong virulence is attributed to its dimorphic ability, where it disseminates at the budding stage, and hyphae aid in its persistence and tissue invasion. Since hyphae are exceedingly too huge to be phagocytosed, extracellular killing by release of NETs is an ultimate strategy to suppress the hyphal form, and numerous studies have revealed that NETs are adequate to kill *C*. *albicans* yeast and hyphae [66].

F. Fan et al. extensively studied the keratitis caused by *Candida albicans* (*C. albicans*) in a mouse model and indicated that contrasted with the non-treated group, the dexamethasone (DXM)-treated group exacerbated the intrusiveness of fungi by subduing the NETs formation [67]. It is fascinating to present new visions of comprehension of the biochemical processes of fungal keratitis and of exploiting the mechanism of NETosis during fungal keratitis. Recently, some efforts to identify the effectors of NETs induction during a fungal infection have unveiled calprotectin as a central antifungal agent in the reaction against *C. albicans* [68]. Calprotectin is a protein that is found in cytoplasm and is released during NETosis. It chelates Mn^2+^ and Zn^2+^, which are crucial for *C. albicans* growth, and thus, immediate contact of the protein with the pathogen is not a prerequisite. Recently, mouse studies have underscored the significance of calprotectin in antifungal defense by showing the high sensitivity of calprotectin-deficient mice towards subcutaneous and pulmonary candidiasis as well as aspergillosis. Furthermore, patients that received gene therapy to reinstate NADPH oxidase function that aids to redeem the efficient NET release were able to recuperate from *Aspergillus* infections much faster in a calprotectin-dependent manner [27,69].

Many controversial reports are available in the literature about the upstream regulators of NETosis inducers in response to fungi. Some reports suggest that conidia induce a little less NETs than hyphae, whereas other reports indicate that *A. nidulans* conidia can be killed more effectively via NETs than its hyphae [27,70]. Even though further studies are necessary to completely appreciate the impact of NETs as the immune shield against fungal infection, there is clear proof that NETs formation is an essential antifungal innate immune strategy.

### 4.3. NETs in Viral Eye Diseasess

Neutrophils are the innate immune cells recruited to sites of viral infections in the most abundance and exhibit both protective and pathologic functions [71,72,73]. In antibacterial and antifungal immunity, the role of neutrophils is well characterized. However, in antiviral immunity, far less is known. Traditional wisdom implies that neutrophils develop antiviral defenses, yet evidence for that is sparse [73]. Interaction with other immune cell populations, virus internalization and killing, and the release of cytokines, chemokines, and antimicrobial components are all mechanisms by which neutrophils can contribute to pathogen clearance. Although conventionally regarded as a defense mechanism against bacterial infections, recent findings have also implicated NET formation in limiting viral infections [7,71,72,73]. These virus-induced NETs can both control the virus and damage the host [7,74]. Recently, HIV- was shown to induce NETosis via the cell death pathway although the requirement for ROS, MPO, and NE was not addressed. NETs are able to seize and nullify the negatively charged HIV virions, substantially decreasing HIV infectivity. Thus, neutrophils and NETs may play critical roles in combating HIV. Interestingly, HIV is efficient in controlling neutrophil activation in order to restrain NET formation.

Saitoh et al. demonstrated that HIV engages DC-SIGN (CD209) on dendritic cells (DCs) with its envelope glycoprotein gp120. Engagement of DC-SIGN leads to production of IL-10 by DCs, which suppresses NET formation. Therefore, HIV not only takes advantage of DC-SIGN on DCs for efficient infection of CD4+ T cells via the DC-T cell synapse but also for evading NET killing [22]. This study remarkably demonstrates an amazing feature of NETs as antiviral effectors and the ability of HIV to coevolve and adapt to the innate immune response. IL-10 is an immunosuppressive cytokine that also impedes TLR-induced ROS production [75]. It is fairly often produced in the milieu of viral infections, suggesting that more viruses exploit IL-10 as a means of NET evasion [76,77]. In the genome of several large DNA viruses, IL-10 homologs have been found, including ubiquitous human pathogens such as human cytomegalovirus (HCMV) and Epstein–Barr virus (EBV) [78,79]. As these virus-encoded IL-10 molecules shape the function and cell death of immune cells, they may also modulate NETosis, similar to cellular IL-10 [80,81].

Finally, the role of NETs in influenza infection has also been investigated. NETs are induced secondarily by influenza-activated lung epithelium producing superoxide and H_2_O_2_. Moreover, while influenza has been indicated to induce NETs in the mouse lung, NET deficiency in PAD4-knockout mice was not linked with an increase in viral titers and susceptibility to infection. Therefore, while few studies have addressed the role of NETs in viral infection, given that many viruses elicit neutrophil recruitment, it is possible that NETs may be implicated in antiviral defense [82]. Additionally, that these viral particles are inactivated is a debatable point; as long as they are ensnared by NETs, they represent no threat. However, an expanding number of studies indicate that a disproportionate virus-induced NET release can contribute to damage locally as well as systemically. Thus, it is important to explore the mechanisms that control NET formation in the context of viral infection [71,72,73].

## 5. NET-Associated Host Damage

Numerous reports suggest the antimicrobial effect of NETosis and its protective role in the host against a range of biological and non-biological disease-causing agents, but its overproduction can be detrimental to the host as well [83]. Multiple reports suggest that dysregulated production of NETs lead to pathogenesis of some metabolic, autoimmune, and autoinflammatory diseases. NETs formation can also cause morbid septic condition. NETs can be extremely cytotoxic to epithelial and endothelial cells due to their constituents such as histones that are antimicrobial, but their unbalanced production can easily cause tissue damage and many pathological abnormalities in eyes [83]. Specifically, during sustained or unwarranted release of proinflammatory cytokines (TNF and IL-1) and the release of toxic bodies such as hydrolytic enzymes, MPO, and ROS, neutrophils can lead to extracellular matrix (ECM) obliteration, massive cell death, and tissue necrosis. The emission of cathepsin G, NE, and proteinase 3 can encourage further vascular leakage, inflammation, and pathologic effects [7]. Moreover, NETs are capable of obstructing secretory ducts, thereby steering higher inflammation [83]. The obstruction of lacrimal duct during an ocular infection can lead to blockage of the drainage system, causing tears to well up on the surface of the eye. Other components of NETs such as anti-high-mobility group box 1 (HMGB1) may also perform a negative role in virus-associated disease [84]. 

The unbalanced NET formation and obstructive clearance leads to circulation of NETs in the serum, which is easily detectable. This systemic NET burst has extreme direct and indirect harmful effects. First, these NETs can damage the interior lining of blood vessels. Second, the flood of the NETs can induce autoantibodies leading to autodestructive processes. NET components such as dsDNA, histones, MPO, vimentin, and enolase have been associated to systemic pathology linked with disease entities such as small vessel vasculitis, systemic lupus erythematosus (SLE), disseminated intravascular coagulation, preeclampsia, and rheumatoid arthritis [85,86].

These systemic consequences justify how NET formation is a part of an antiviral defense strategy and acts as a double-edged sword. The host may gain from deposition of NETs, specifically in the infected area by restraining and nullifying the virus and finally killing the virus-infected cells. This advantage may turn into a fiasco if NET formation is overly spread, establishing NET deposits in healthy tissues. As a result, too many uninfected host cells in the neighborhood of the infected area may come under “friendly fire”, leading to substantial collateral tissue damage. Thus, it is important to study the role of NETosis in host defense as well as in disease severity (Table 1).

## 6. Diagnosis and Therapeutics

NETs can be both beneficial and destructive for the host; thus, it is important to evaluate possible compounds that specifically suppress NETs. Likewise, it is equally important to positively detect the NETs (Table 2). The markers of NETosis can be prospective drug targets to treat infection. Given evidence of the association of NETosis with several disease pathologies, there are two approaches to mitigate its effects. The first is to include the use of drugs that suppresses NETosis, such as anti-cytokine therapy targeted to avoid neutrophil buildup in the foci and their initiation, as well as employing inhibitors of the elements engaged in NETosis system: NE, PAD4, and GSDMD. The second methodology is centered on attenuating the negative effects of NETosis. It constitutes anti-cytokine therapy aimed against IL-1β and is extensively applied in numerous inflammatory and autoimmune diseases [92].

Our implication is that most of the NETosis targeted inhibitors will show their potential effectiveness against ocular herpetic infection as well. For instance, the NADPH- and/or ROS-inhibiting pharmacological compounds such as diphenyleneiodonium chloride (DPI) and *N*-acetylcysteine (NAC) suppress NETosis, and many reports suggest reduction in ocular herpes infection when treated with antioxidants because it has been reported that HSV infection may be exacerbated by a cellular state of oxidative stress, while augmented intake of antioxidants might avert replication of the virus [100,101,102,103,104]. Furthermore, ROS inhibit NF-kB (Nuclear Factor kappa-light-chain-enhancer of activated B cells) translocation, which is a known marker for aggravated ocular HSV-1 infection [105]. Anti-cytokine therapy addressed against IL-1β is extensively utilized in several inflammatory and autoimmune diseases, including ocular herpetic disease. One of its aims may be unwarranted NETosis. The recombinant anakinra protein, an IL-1β receptor antagonist, is at present under clinical trials as a prospective formulation to cure COVID-19 (NCT04324021, NCT04330638, and NCT02735707). This drug might as well find its implication in ocular herpes pathology [92]. Finally, a few GSDMD and PAD4 inhibitors are at the preclinical stage of testing as well. Of great attention and interest is the application of present-day drugs for suppression of NETosis. Hence, disulfiram, which is clinically utilized to treat alcoholism, inhibits GSDMD activation and shields mice in the lethal LPS-induced sepsis model [106]. It is worth noting that GSDMD is also a key element of pyroptosis, and many authors have acknowledged the potential role of pyroptotic pathway during an HSV-1 infection [107,108,109,110]. Furthermore, there is also a cell-intrinsic program that modifies the neutrophil proteome in the circulation and causes the progressive loss of granule content and reduction of the NET-forming capacity [111]. This program is driven by the receptor CXCR2 and by regulators of circadian cycles. Indeed, it is also known that HSV-2 infection is influenced by circadian cycle [111]. The HSV-2 entry receptor Nectin1 (*Pvrl1*) in mouse and human keratinocytes shows rhythmic expression and is directly regulated by CLOCK, and HSV-1 infection varies with the disruption of the transcription factor BMAL 1 (brain and muscle ARNT-like 1) [112,113]. Several studies have demonstrated the importance of the circadian parameter in clinical settings, which raises the possibility of using these temporal physiological features for therapeutic benefit (i.e., chronotherapy). These findings highlight the possibility of “personalizing” medicine at the temporal level [114].

## 7. Conclusions

Neutrophils are recruited as the first line of host defense during an ocular infection. They respond to corneal-surface chemo-attractants and help perform effector functions such as phagocytosis during an ocular HSV-1 infection. Two decades ago, the role of neutrophils in the formation of NETs and NETosis were reported, and since then, it has been widely studied in microbial infections. Still, very little is known about NETosis in viral infections of the eye. Evidence exists showing that HSV-1 infection of the eye is influenced by NET formation, which may help reduce the viral burden but may also cause an increase in the tissue inflammation. Many doubts and open-ended questions surround the fundamentals of NETosis itself. In particular, the targets for induction of NETosis leading to activation of NADPH oxidase have not yet been clearly determined. It is evident that excessive NETosis can lead to many inflammatory pathologies and in turn harm the host. Given the significance of neutrophils in ocular HSV-1 infection, a new push to investigate the pathophysiological role of NETosis will improve our understanding of the pathogenesis of ocular HSV-1 infection. The development of new therapeutics inhibiting NETosis, majorly targeting ROS, is likely to be a promising area of future pharmacological research in curbing HSV-1 infection and associated ocular pathologies.

## Figures and Tables

**Table 1:** NETosis in viral infections.

Pathogen	Induction of NETosis	Prognosis	References
Influenza A virus (H1N1) PR8 strain	Extensive NETs induction when neutrophils were incubated in the presence of H_2_O_2_, which may in turn activate MPO and cause NET formation.	Excessive NET formation after H1N1 infection contributes to acute lung injury and acute respiratory distress syndrome (ARDS).	[87]
Influenza A virus	Infected patients showed higher capacity to release MPO-DNA complex in response to interleukin-8 or lipopolysaccharide stimulation.	NETs from infected patients increased the permeability of alveolar epithelial cells and consequently caused acute physiology and chronic health evaluation (APACHE) II score and MODS.	[88]
Human immunodeficiency virus-1 (HIV-1)	Activation of endosomal TLR7- and TLR8-mediated NET formation.	NETs captured HIV-1 and promoted its elimination by inhibiting its infection and spreading.	[22]
Human respiratory syncytial virus (RSV)	Chemokines such as interleukin-8 (IL-8) are abundantly present in the lungs during RSV–LRTD	NETs trap viral particles in vitro, but their exaggerated formation during severe RSV–LRTD contributes to airway obstruction in children.	[89]
Hantavirus (HTNV)	Heparin-sensitive β2 integrin receptors are involved in hantavirus-induced generation of ROS and NETs.	NETs are detected in kidney biopsies from hantavirus-infected patients, suggesting that NETs contribute to kidney damage.	[90]
SARS-CoV-2	The N-terminal (GSDMD-NT) oligomerizes with plasma and nuclear membranes, forming membrane pores that mediate cell death by NETosis.	Lung epithelial damage and disseminated intravascular coagulation.	[91]

**Table 2:** Techniques and markers for NETosis detection.

Technique	Reagents	Markers	Limitation	References
Immunostaining for neutrophil-derived proteins	Acetone, ethanol, and paraformaldehyde (PFA)	MPO and proteinase 3 (PR3)	Acetone and ethanol can induce an artificial NET formation. Qualitative and lacks objectivity.	[93]
Immunostaining	Acetone and ethanol	Citrullinated histones produced due to PAD4-mediated NETosis	The involvement of PAD4 in NET formation depends on the nature of the stimulation and remains debatable.	[94,95,96]
Fluorospectrophotometry	PicoGreen	Cell-free DNA or neutrophil remnants in sera or tissue fluids	Cell-free DNA does not originate specifically from netting neutrophils. They can be derived from dead cells other than neutrophils that undergo NETosis.	[97]
Enzyme-linked immunosorbent assay (ELISA)	Horseradish peroxidase (HRP)-conjugated anti-DNA antibody	MPO-DNA and NE-DNA complexes in fluid samples	Standardization in ELISA remains elusive, but this methodology is the current most specific, objective, and quantitative assay to monitor NETosis.	[93]
Multispectral imaging flow cytometry	2% PFA solution and Hoechst for nuclear labeling and stains MPO	Images the increase in the nuclear area, which coexists with the decrease in side-scatter intensity of the cells or with overlapped distribution of MPO.	Further studies are needed.	[98]
Flow cytometry	SYTOX Green	Cell-appendant DNA of netting neutrophils.	More extensive studies are needed to determine if this method can distinguish NETosis from other types of cell death.	[99]

## Data Availability

Not applicable.
